# Bioinformatics Resources and Tools for Conformational B-Cell Epitope Prediction

**DOI:** 10.1155/2013/943636

**Published:** 2013-07-21

**Authors:** Pingping Sun, Haixu Ju, Zhenbang Liu, Qiao Ning, Jian Zhang, Xiaowei Zhao, Yanxin Huang, Zhiqiang Ma, Yuxin Li

**Affiliations:** ^1^School of Computer Science and Information Technology, Northeast Normal University, Changchun 130117, China; ^2^National Engineering Laboratory for Druggable Gene and Protein Screening, Northeast Normal University, Changchun 130024, China; ^3^Key Laboratory of Intelligent Information Processing of Jilin Universities, Northeast Normal University, Changchun 130117, China

## Abstract

Identification of epitopes which invoke strong humoral responses is an essential issue in the field of immunology. Localizing epitopes by experimental methods is expensive in terms of time, cost, and effort; therefore, computational methods feature for its low cost and high speed was employed to predict B-cell epitopes. In this paper, we review the recent advance of bioinformatics resources and tools in conformational B-cell epitope prediction, including databases, algorithms, web servers, and their applications in solving problems in related areas. To stimulate the development of better tools, some promising directions are also extensively discussed.

## 1. Introduction

A B-cell epitope is defined as a region of an antigen recognized by either a particular B-cell receptor (BCR) or subsequently the elicited antibody in a humoral response [1–3]. A B-cell epitope can be categorized into two types by its spatial structure: liner epitope or conformational epitope. A liner epitope (also called continuous epitopes) is composed of residues that are sequentially consecutive, whereas a conformational epitope (also known as discontinuous epitope) consists of sequential segments that are brought together in spatial proximity when the corresponding antigen is folded. It has been reported that more than 90% of B-cell epitopes are discontinuous B-cell epitopes [4, 5].

The identification of B-cell epitopes is rather important to immunodetection and immunotherapeutic applications since an epitope as the minimal immune unit is strong enough to elicit a potent humoral immune response with no harmful side effects to human body [3, 6]. The ultimate goal of epitope prediction is to aid the design of molecules that can mimic the structure and function of a genuine epitope and replace it in medical diagnostics and therapeutics and also in vaccine design [2, 7]. The most reliable methods for identification of an epitope are X-ray crystallography and NMR techniques [8, 9], but they are time consuming and expensive. Hence, computational methods and tools, with the virtues of low cost and high speed, were employed to predict B-cell epitopes in silico.

The interaction between an antigen and an antibody is a complicated biochemical process. An antibody, which has a “Y”-shape structure, binds to the epitopic region of an antigen through a highly variable complementarily determining region (CDR). The interaction between an antigen and an antibody is mainly through the connections of intermolecular low energy (e.g., hydrogen bond, hydrophobic interaction, and van der Waals force) and few connections of intermolecular high energy (e.g., salt bridge). Moreover, since an antibody interacts with an antigen through a deep and narrow antigen-binding clef, it is reasonable to believe that the interaction between an antigen and an antibody involves both specific sequence recognition and mutual structure identification.

By far, the study of B-cell epitope prediction mainly aimed at predicting linear epitopes [10–24]. However, since most B-cell epitopes are conformational epitopes, the prediction of liner B-cell epitope has limited application. In recent years, some computational methods were proposed though the number is limited and the performance is not significant [25–29]. Consequently, to improve the performance of B-cell epitope prediction, integrating multidisciplinary knowledge and combining different methods become a promising prospective.

In this work, we review recent advances in computational methods for conformational B-cell epitopes prediction, including databases, algorithms, web servers, and their applications, point out some problems in the current state of the art, and outline some promising directions for improving the prediction of conformational B-cell epitopes.

## 2. Structure-Based Prediction Methods

B-cell epitopes prediction based on the 3D structure of antigen began in 1999 [30], and the core idea of the prediction methods is through the 3D structure of antigen and epitope-related propensity scales, including geometric attributes and specific physicochemical properties. In recent years, with the development of various omics and bioinformatics, related experimental data of conformational B-cell epitopes has been accumulating rapidly. The development of epitope-related databases promotes conformational B-cell epitopes prediction. Herein, we review the major databases and approaches for predicting conformational B-cell epitopes based on the 3D structure of an antigen.

### 2.1. Databases

The availability of experimental data plays a pivotal role in conformational B-cell epitope prediction. The 3D structure of antigen or the complex of antigen-antibody is stored in the PDB database [31], and the data for epitopes and other associate information were stored in some special databases.  Table 1 lists all the epitope-related databases together with their functional comments. 

PDB [31] database compiles the compounds derived from the X-ray crystallography and NMR experiments. The majority of the information from PDB database are the 3D structure of protein. One can search needed structure according the PDB-id in the home page and then view or download the structure in several formats. CED database [32] comprising the annotated epitopes which was determined by experimental methods. The database provides a user-friendly web interface, and most epitopes in database can be viewed interactively in the context of their 3D structures. One can browse all the entries or search the certain entry from the corresponding hyperlinks in the home page. IEDB database is the most commonly used and most authoritative database in epitope prediction [33, 34]. Since IEDB 2.0 released, there were 38,552 entries on B-cell epitope and a handful of integrated prediction tools providing much convenience for researchers. Researchers can search interested B-cell epitope from the pull-down menu of “Advanced search” on the home page. HIV Molecular Immunology database contains HIV virus epitopes which were determined by experiments [35]. Both the B-cell epitopes and T-cell epitopes are included. This database provides convenience for the research of specific HIV virus epitopes.

The previous databases are important resources for conformational B-cell epitope prediction. The data from these databases provide a basis for computational biologists to derive benchmark and customize datasets for new algorithm development and tool evaluation. 

### 2.2. Algorithms, Programs, and Their Application

Comparing with mimotope-based prediction methods which will be introduced in what follows, structure-based methods for conformational B-cell epitopes prediction have the advantage that they only need the structure of antigen. In 1999, Kolaskar and Kulkarni-Kale used the 3D structure of antigen to analyse and locate the conformational epitopes of Japanese encephalitis virus by calculating the surface accessible fragments of amino acids [30]. They improved the algorithm and released CEP which is the first web-based software for conformational epitope prediction in 2005 [36]. The essential ideal of CEP is to generate surface fragments of an antigen, and then use the spatial distance of these fragments and other statistical characteristics to locate epitopes. The structure-based algorithms, web servers (programs) and brief notes are listed in Table 2.

DiscoTope was the second web-based conformational epitope prediction software [37]. In 2006, Andersen et al. collected a dataset which contains 76 antigen-antibody complexes. To investigate the role of certain features that distinguish epitopes from nonepitopes, a number of statistics were studied including the distribution of length and segments of an epitope and single amino acid preference and Parker hydrophilicity. Through a combination of statistics, spatial context, DiscoTope could successfully predict the location of epitopes on the previously mentioned dataset. In 2007, Rapberger et al. proposed a new kind of conformational B-cell epitopes prediction framework [38]. They took advantage of the complementary geometric shape of antigen epitopes and antibody paratope, as well as the measure of binding energy of antigen and antibody. The method was the first one which considered the antibody information in the research of epitopes prediction.

The first conference for B-cell epitope prediction was held in Washington 2007. The meeting published a benchmark dataset for conformational B-cell epitope prediction in the format of the 3D structure of antigen chosen from PDB database. The benchmark dataset includes 62 3D structures of antigens with inferred epitopes. The construction of this benchmark dataset accelerated the development of conformational B-cell epitopes prediction and provided a basis for method evaluation. Ponomarenko and Bourne evaluated CEP and DiscoTope using the benchmark dataset in the same year [39]. The results indicated that the performance of both methods did not exceed 40% of precision and 46% of recall. Consequently, methods with better performance are still in great need. One way to attain this goal is through developing new features and combining them.

In the next few years, newly proposed conformational B-cell epitope prediction methods managed to look for effective propensity scales or combine the available amino acid physicochemical properties and geometrical structure properties. In 2008, three conformational B-cell epitope prediction methods were proposed: ElliPro [40], PEPITO [41], and PEPOP [42]. The main idea of ElliPro attributes to the liner B-cell epitopes prediction method of Thornton et al. [43]. ElliPro predicts conformational B-cell epitopes by combining the geometric features of an antigen and single amino acid epitope propensity. When the structure is not available, ElliPro first model the 3D structure of the antigen by searching for its homologues in PDB or running MODELLER [44]. PEPITO predicts conformational B-cell epitopes using a combination of single amino acid epitope propensity and half sphere exposure values at multiple distances. One major improvement of PEPITO is that it employed half sphere exposure to describe the degree of compactness which inspired the latter methods. PEPOP identifies segments composed of accessible and sequentially contiguous amino acids of the 3D structure of an antigen and then clusters these segments according to their spatial distances to identify epitopes. Another contribution of PEPOP is designing immunogenic peptides through the results of epitopes identification.

SEPPA [45], Epitopia [46], and EPCES [47] were published in 2009. SEPPA employs the concept of “unit patch of residue triangle” to describe the local spatial context of protein surface and “clustering coefficient” to describe the spatial compactness of surface residues. Then, the two features are combined to predict epitopes. Epitopia adopts the idea of partition which divides a given antigen to overlapping surface patches. Then, the scores of physicochemical and structural-geometrical properties for central residue of each patch are calculated before using a Naïve Bayes classifier to predict the immunogenic potential of protein regions. EPCES proposed six epitopes propensities, including conservation score, side-chain energy score, contact number, surface planarity score, and secondary structure composition. With the vote mechanism, EPCES reaches a consensus score which represents the likelihood of being an epitope based on the scale of each feature. Based on the features, we trained an SVM classifier to predict conformational epitopes [48], and the testing results showed that different classification methods did not improve the accuracy of the prediction performance based on these propensities.

To develop better features, Soga et al. emphasized information hidden in antibody in the process of antigen and antibody interactions [49]. They defined the antibody-specific epitope propensity (ASEP) index. Then, it was used to predict epitopes together with the result from DiscoTope. This paper made the first attempt to identify epitopes by combining different prediction methods. In 2011, Sun et al. collected a latest comprehensive dataset and did detailed statistical analysis of epitope residues and nonepitope residues from several aspects [50]. The study of antigen and antibody interaction pattern revealed the importance of antibody information in epitopes prediction as well. In the same year, two novel server applications EPSVR and EPMeta were presented by the same author of EPCES [51]. EPSVR uses a support vector regression method to integrate six scoring terms as EPCES, while EPMeta is a metaserver which combined with EPSVR, EPCES, Epitopia, SEPPA, PEPITO, and Discotope1.2. In 2011, Zhang et al. proposed a new epitope prediction method [52]. The method proposed a concept of “thick surface patch” which brought the impact of interior residues, the adjacent residue distance feature, into consideration. It reflects the unequal contributions of adjacent residues to the location of binding sites and the random forest algorithm which is used to process imbalanced data. The method represented higher prediction accuracy comparing with other methods.

The structure-based conformational B-cell epitopes prediction methods are all based on the structure features of antigen, and a different method employs different propensity scales. Most of the previously mentioned prediction methods offer online service or program (see Table 2). The online services have a user-friendly interface. The usage of these methods is simple. Researchers enter the PDB ID or upload the local file in PDB format, determine the antigen chain, and specify the corresponding thresholds according to the orders that will later get the prediction results. Yao et al. [53] construct a benchmark and evaluate the performance of all existing prediction methods. The results show that the accuracy of EPMeta is the overall highest value by all conditions and methods. It states that in the case of different prediction methods usually not give a consensus result, and consider the results of the multiple prediction methods is a better choice.

### 2.3. Current Problems

B-cell epitope prediction based on the 3D structure of antigen structure has already made some progress, even so the methods need further improvements. Firstly the dataset, which is essential for the methods based on machine learning, is relatively small and inconsistent. Moreover, since nonepitopic amino acids are defined as the amino acids which are not a part of currently determined epitopes, the undetermined epitopic amino acids would very likely bring in noises in the process of statistical learning. In addition, the input and output formats for each method is different which make it difficult to evaluate the performance of different methods.

Secondly, in order to assess the validity and performance of the prediction methods, both antigen structure and the epitope information are needed. CED and IEDB annotated epitope sites for part of structures, and we call this annotated epitopes which are actually determined by wet experiment as functional epitopes. But this situation is not the same for the other structures. To use these structures, one needs to determine the epitope of the structures by distance between antigen and antibody or accessible surface area ((ASA), and Surface Racer [54] and NACCESS [55] are commonly used tools that are designed for calculating ASA) loss upon antibody binding at first, and we call this kind of epitope as structure epitopes. The difference in epitopes determination makes prediction methods producing relatively poorer performance on the structure epitopes-based datasets than on the functional epitopes based datasets. 

Lastly, an antibody binds to an antigen by the spatial structure, so there is a wealth of information hidden in the 3D structure of antigen and antibody. Theoretically, the features extracted from the structure would certainly improve the performance of existing B-cell epitopes prediction methods. However, it is more complicated to extract features from the 3D structure of an antigen than dealing with the primary sequence. Features mentioned in these papers do not have enough ability to distinguish the epitopic residues from the rest.

## 3. Mimotope-Based Prediction Methods

Mimotope-based prediction is a combinatorial method which requires both antibody affinity-selected peptides and the 3D structure of antigen as input. To attain affinity-selected peptides, random peptides are initially displayed on the surface of filamentous phages. Then, random peptides which bind to a monoclonal antibody with a certain degree of affinity are screened, eluted, and amplified. After 3–5 rounds of the operation, the resulting peptides become fewer but with higher affinity. These affinity-selected peptides are defined as mimotopes. Mimotopes and genuine epitopes can combine the same paratope of monoclonal antibody and cause immune response, so they have the similar functionality with the genuine epitope [56, 57]. Besides, the selected mimotopes commonly share high sequential similarity which implies that certain key binding motifs and physicochemical preferences exist during the interaction. Therefore, mapping these mimotopes back to the source antigen can help finding the genuine epitopes more accurately. In what follows, we review the major databases and approaches for predicting conformational B-cell epitopes based on mimotopes.

### 3.1. Databases

Mimotope-based methods need both the structure of antigen and the sequence data of mimotopes. Since the 3D structure of the antigen can be obtained from PDB or by computational homology modeling, the small number of mimotope sequences derived from phage display becomes a limitation for the development of conformational B-cell epitopes prediction based on mimotopes. In recent years, several databases which integrated the structure data, the mimotopes data, and other associate information have been released which play a fundamental role in Immunoinformatics.  Table 3 lists current databases which contain the information of mimotope.

ASDP was a curated database that incorporated data on full-length protein, proteins, protein domains, and peptides which were obtained mainly from phage display experiment [58]. It was the first database for mimotopes. The current version released in 2001 has 195 entries. ASPD has a user-friendly interface, and researchers can search the needed information by means of the SRS system. The RELIC Peptides is a relational database that contains more than 5,000 peptide sequences selected with small molecule metabolites drugs as well as random clones from parent libraries [59]. RELIC Peptides is indispensable as part of the RELIC suite for many tools in RELIC depend on the data. PepBank is a database of peptides based on sequential text mining and public peptide data sources [60]. This database stores peptides with available sequences and the length equals 20 amino acids or shorter. PepBank has a web-based user interface with a simple, Google-like search function, advanced text search, and BLAST and Smith-Waterman search capabilities. MimoDB is an information portal to biopanning results of random libraries [61, 62]. It is the latest and largest database for mimotopes. In version 2.0, it has 15,633 peptides collected from 849 papers and groups into 1,818 sets. For each entry, the target, template, library, and structures information are given. In addition, MimoDB provides tools for simple and advanced search, structure visualization, BLAST, and alignment view on the fly. 

Sun's benchmark datasets were constructed by our team in 2011, and it is special for conformational B-cell epitope prediction based on mimotope analysis. Now, we have established benchmark 2.0 already. The benchmark 2.0 consists of 39 complex structures with 66 mimotope sets; the 39 complex structures contain 16 antigen-antibody complexes and 23 protein-protein interactions structures. In addition, we provide 24 test cases as representative datasets which have only one mimotope set for one complex structure. Each set includes the complex structure, the template chain, the mimotopes obtained from corresponding phage display experiment, and the epitope information. All the datasets can be downloaded freely for academic purposes. The benchmark dataset can be freely accessed at http://cs.nenu.edu.cn/bioinfo/benchmark%20datasets/index.html.

 The databases described previously are important resources for the mimotope-based B-cell epitope prediction. With the large amount of mimotopes in these databases as well as the protein structure databases, it is feasible to construct a benchmark for development and evaluation of new mimotope-based epitope prediction methods.

### 3.2. Algorithms, Programs, and Their Application

Mimotope-based prediction methods are essential to map mimotopes back to the surface of a source antigen to locate the best alignment sequences and predict possible epitopic regions. The available mimotope-based algorithms, web servers (programs), and brief notes are listed in Table 4.

Huang et al. classified the mimotope-based epitope prediction into two categories: one is the sequence-sequence alignment methods and the other is sequence-structure alignment methods [63]. Among the prediction methods listed previously, FINDMAP, EPIMAP, and the MimAlign algorithm of MIMOP belong to the sequence-sequence alignment methods. The inputs of these methods are mimotopes and the primary structure of an antigen. FINDMAP aligns the motif extracted from mimotopes to the antigen sequence directly rating the best matching sequences as epitope candidates [64]. EPIMAP is an improved version of FINDMAP [65]. It aligns each mimotope to the antigen sequence and then selects the most mutually compatible alignments from a set of the top-scoring alignments before filtering out spurious alignments with EPIFILTER program. MIMOP was proposed by Moreau et al. in 2006 [66] which includes two parts: MimAlign and MimCons. MimAlign combines results from four multiple sequence alignments of the antigen and mimotopes sequences in a combined alignment. For each position of the combined alignment, a frequency and a score are calculated. Convergent positions are then selected and clustered based on their topology. The clusters attained are considered as potential epitopic regions.

The remaining methods belong to the sequence-structure alignment methods. Further, Huang classified these methods into 5 kinds according to the mean of sequence-structure alignment [63]: motif-based methods, pairs-based methods, patch-based methods, graph-based methods, and hybrid methods.

The motif-based methods aim to obtain motif through multiple alignment of mimotopes and then map the motif to the surface of an antigen to locate B-cell epitopes. MEPS, 3DEX, MIMOX, and the MimCons algorithm of MIMOP belong to this kind. MEPS is the first B-cell epitope predicting method based on mimotope analysis [67]. MEPS first model an antigen surface into fixed-length peptides and then aligns each of the short peptide to the motif derived from multiple alignment of the mimotopes. The best aligned short peptides are treated as candidate epitopes. 3DEX takes the physicochemical neighborhood of C*α*- or C*β*-atoms of individual amino acids into account [68]. A given amino acid in a peptide sequence is localized by the protein, and the software searches within predefined distances for the amino acids neighboring that amino acid in the peptide. Surface exposure of amino acids can also be taken into consideration. The procedure is then repeated for the remaining amino acids of the peptide. This procedure may cost few hours. MIMOX is the first freely accessible web tool for mimotope-based B-cell epitope prediction [62]. It has two parts. The first part provides a simple interface for the alignment of mimotope sets, while the second part of MIMOX maps a single mimotope or a motif derived from the first part onto the corresponding antigen and rates all of the clusters of residues to locate the genius epitope. MimCons is another part of MIMOP method, and it evaluates the similarity of the mimotope sequences and clusters them accordingly. Motifs are identified from mimotope sequences of each cluster. The accessible surface of the antigen is scanned to find out all possible exposed consensus patterns. Spatial neighbor amino acids are identified and constitute potential epitopes. In addition, MimAlign and MimCons can be run either independently or with their results combined.

The essential idea of pairs-based methods is to predict B-cell epitopes with the statistical characteristics of amino acid pairs. Mapitope and Denisova belong to this kind. In 2003, Enshell-Seijffers et al. described a mimotope-based approach to predict the epitopes of the HIV-1 [69]. Firstly, they defined amino acid pairs (AAP) with a predefined distance threshold between the central carbon atom of two neighbor residues. Secondly, they defined statistically significant pairs (SSPs) by calculating the probabilities of each AAP. Lastly, the SSPs are mapped to the 3D structure of an HIV-1 antigen to locate epitopes. In 2007, Bublil et al. applied this method to conformational B-cell epitope prediction and presented the tool as Mapitope [70]. A continuous work by Denisova et al. took all possible space pairs, including pairs separated by one residue, two residues, three residues, and so on in mimotopes into account and identified epitopes by pattern recognition theory [71–73]. This method is specially designed for elucidating epitope specificity within antiserum. 

The core idea of patch-based methods is dividing the surface of antigen into overlapping patches and selecting high-scored amino acid residues as candidate epitopes by comparing mimotopes with patches based on sequence similarity or the statistical characteristics of amino acids. SiteLight and EpiSearch belong to this category. SiteLight divides the antigen surface into overlapping patches, and then aligns each mimotope to each of the patches. To identify candidate epitopes, the best matched paths are selected repeatedly until 25% of antigen surface is covered [74]. EpiSearch predicts conformational B-cell epitopes by an automated sequence analysis of mimotopes and a comparison to the distribution of amino acids on patches on the antigen surface [75]. The amino acid compositions of the mimotopes and 3D profile of an antigen are compared and quantified in a score function for each patch on the antigen surface. The highest scoring patches are listed in the output files and are also displayed on the surface of the protein.

The main idea of graph-based methods is to model the amino acids from an antigen as a graph structure so as to use the graph search methods to locate potential epitopes. PepSurf and Pep-3D-Search belong to this category. PepSurf searches the best matched paths from the graph built from the antigen with mimotope sequences using color-coding algorithm and dynamic programming algorithm [76]. Pep-3D-Search searched for the matched paths on the antigen surface by the Ant Colony Optimization (ACO) algorithm [77]. Candidate epitopes were then formed by clustering the resulting paths with a high *P* value score by the Depth-First Search algorithm. Pep-3D-Search provides two modes of B-cell epitope prediction: (1) mimotope-based search and (2) motif-based search.

The last kind of mimotope-structure alignment B-cell epitope prediction method is a hybrid method. MimoPro, which was proposed by our team in 2011, is the first attempt to integrate the idea of different methods. The method employs the idea of patch-based and graph-based searching [78]. The core of MimoPro is a searching algorithm operated on a series of overlapping patches on the surface of antigen. These patches are then transformed to a number of graphs using an adaptable distance threshold (ADT) regulated by compactness factor (CF), a novel parameter proposed in this method. Then on each single patch, a complete search is conducted to guarantee the best alignment for each mimotope sequence. Dynamic programming and branch-bound methods are also adopted to both avoid repetition in searching and further narrow the search space.

Unfortunately, the available service of the previous 14 methods is few. At present, there are only three available freely web-based B-cell epitope prediction service platforms in the world. The first is PEPITOPE [79], and it provides online service based on three methods: Mapitope, PepSurf, and the combined. The web service of the three methods has the restriction that the length of mimotope sequence cannot be longer than 14 amino acids. Besides, Mapitope and PepSurf also can be run in local, and the local version has no service restriction. The second is EpiSearch and the epitope prediction method is EpiSearch only [75]. EpiSearch has the restriction that the number of mimotope sequences cannot exceed 30 amino acids. The third prediction platform is PepMapper which is released by our team in May 2012 [80]. PepMapper also provides online service based on three methods: Pep-3D-Search, MimoPro, and the combined. Since Pep-3D-Search is based on the establishment of empirical background distribution for aligning score of every mimotope and antigen, and if the *P* value of aligning score for every mimotope is bigger than 10^−3^, Pep-3D-Search will not give any prediction result. Among all these methods, only MimoPro has no limitation. As the structure-based conformational B-cell epitopes prediction methods, a different method employs different prediction strategy, and will not give a completely consensus prediction result. As Liang's idea [53], we think meta-analysis may be a better solution, and we are engaged in certifying this idea now.

### 3.3. Current Problems

Mimotope-based B-cell epitope prediction methods located epitopic region through the information from mimotopes which is obtained from experimental methods. Mimotope-based prediction is statistically more accurate, but it requires the information of mimotopes from experimental data. However, comparing with X-ray crystallography and NMR methods, in vitro screening methods have a low price to pay. Moreover, the methods can locate the interacting epitope in a designate antigen-antibody interaction context. 

Despite that, accurate prediction of epitopes is still a long way to go. In 2011, we constructed a benchmark dataset for conformational B-cell epitope prediction and evaluated five mimotope-based prediction software products [25]. The result showed that in no method did the performance exceed a 0.42 of precision and 0.37 of sensitivity. The poor performance of the prediction is rooted in several aspects. The size and diversity of the benchmark dataset is inadequate, as well as many problems in mimotope-based B-cell epitope prediction need to be further studied. MimoPro combines the idea of different methods. By employing a novel idea of ADT which reflects the flexibility of interaction between amino acid pairs, MimoPro reached is the highest sensitivity among the methods, but the overall performance is still not satisfactory. How to express conformational changes in the interactions of antigen and antibody, how to establish rational mathematical model through integration of mimotopes information and the statistical characteristics of amino acids, and design intelligent search algorithm on the surface of antigen are the main directions to further improve the performance of mimotope-based B-cell epitope prediction methods.

## 4. Other Methods

In this section, we will focus on the development of other conformational epitope prediction methods aside from the structure-based methods and the mimotope-based methods.

### 4.1. Sequence-Based Methods

Sequence-based prediction methods only rely on the primary sequence of an antigen and inherit the idea of liner B-cell epitopes prediction. Particularly, the methods employs propensity scales to measure the probability of each residue being part of epitopes [37]. To reduce fluctuations, sliding window strategy is usually used. 

In 2010, Ansari and Raghava proposed a method to predict conformational B-cell epitopes from the primary sequence of antigen [81]. In the method, sparse encoding scheme (BPP), physicochemical features (PPP), and amino acid composition (CCP) are extracted from the overlapping amino acid segments sliced from antigen sequences and used to train a SVM for prediction. There are two newly published methods that predict conformational B-cell epitopes by antigen sequence in last year. The two methods are BEST [82] and Zhang's [83] method. They all extract enough sequence characters first, and then BEST method employed SVM for classification, while Zhang's method adopted the ensemble learning approach to handle various features for epitope prediction.

As the high experimental requirements for resolution of protein 3D structure, the 3D structure of a large number of protein has not been resolved, and the B-cell epitope prediction methods based on antigen sequence may be worth more deeper research. Compared with structure-based prediction methods, the performance of sequence-based methods did not improve a lot, but the thought of sequence-based methods provides innovative research ideas for conformational B-cell epitope prediction.

### 4.2. Binding Sites Prediction Methods

The interaction of an antigen and an antibody is a subtype of protein-protein interaction, so some methods that focus on binding sites prediction of protein-protein interaction can be borrowed for conformational B-cell epitopes prediction. Recently, Yao et al. [53] construct a benchmark and evaluate the performance of all existing structure-based B-cell prediction methods, along with 4 binding sites prediction methods: ProMate [84], ConSurf [85], PINUP [86], and PIER [87]. The results showed that the performances of the binding site prediction methods to predict B-cell epitopes are significantly lower than all structure-based epitope prediction methods. In fact, the interaction between antigen and antibody is different from other kinds of protein-protein interaction in some degree. For instance, protein-protein binding sites are usually more conserved than other surface residues to maintain the functionality of the protein, while the antigen-antibody binding sites (epitope) are less conserved due to the competition for survival against the host immune system. Hence, using these prediction methods for epitopes prediction has certain drawbacks. More importantly, the prediction methods need both the antigen and antibody structure, but epitope prediction methods are designed to identify the potential epitopes on the antigen when the antibodies are unknown. However, the epitope prediction of unbound structure has more practical value in general. Due to the different purposes, the binding sites prediction methods have little advantage in epitope prediction. 

## 5. Conclusions and Prospects

B-cell epitope prediction is important for vaccine design, development of diagnostic reagents, and interpretation of the antigen-antibody interactions on a molecular level. In recent years, with the development of various omics and bioinformatics, related experimental data of conformational B-cell epitopes has been proposed rapidly. The construction of relevant databases promote the development of conformational B-cell epitopes prediction. In this study, we make a systematic review about the bioinformatics resources and tools for conformational B-cell epitope prediction. Though the developments, the overall performance is still not satisfactory. In what follows, we point out several aspects that may improve the performance of conformational B-cell epitopes prediction.

*Build Large and Reliable Datasets.* Areliable dataset should meet the requirement of nonredundant antigen structures (bound or unbound), well-defined B-cell epitopes, and the mimotope sequences. Nonredundant and abundant datasets could avoid the performance of B-cell epitope prediction methods overly optimistic. Well-defined B-cell epitopes is the premise of epitope relevant feature extraction and directly impacts the prediction performance. Mimotopes sequence is especially important for the mimotope-based conformational B-cell epitope prediction. Furthermore, large and reliable datasets are important for both training and testing. Training datasets are used to feature extraction and model training, while testing datasets is responsible for testing the performance of prediction method and evaluating the performance between different methods.

*Extracting Effective Epitope Relevant Features.* The essence of structure-based conformational B-cell epitope prediction is pattern classification. Extracting effective epitope relevant features is the most important part in structure-based conformational B-cell epitope prediction methods which is also the key point in B-cell epitope predictions. By far, there is no single feature or combination of features that can effectively distinguish epitopes from nonepitopes. To improve the performance of conformational B-cell epitope prediction methods, selecting effective features, or feature combination as well as integrating the mimotope-based methods may be a promising area.

*Devise Intelligent Searching Algorithms.* The essence of mimotope-based conformational B-cell epitope prediction is searching similar sequences with mimotopes on the surface of antigen. Intelligent searching algorithms could improve the effectiveness of the methods, as well as the prediction performance.

## Figures and Tables

**Table 1:** Databases for 3D structure of the antigen and epitopes data.

Databases	Websites
PDB [31]	http://www.rcsb.org/pdb/home/home.do
CED [32]	http://immunet.cn/ced/
IEDB [33]	http://www.immuneepitope.org/
HIV Molecular Immunology [35]	http://www.hiv.lanl.gov/content/immunology/index

**Table 2:** Methods for structure-based B-cell epitopes prediction.

Method	Online service (program)	Brief notes (features used in prediction method)
CEP [30, 36]	Available upon request	First Web-based conformational B-cell epitopes prediction software, based on the surface accessible
DiscoTope [37]	http://www.cbs.dtu.dk/services/DiscoTope/	Amino acid statistics, spatial context, and surface accessibility
Rapberger^a^ [38]	Not stated	Based on antibody information
ElliPro [40]	http://tools.immuneepitope.org/tools/ElliPro/iedb_input	Prominent index
PEPITO/BEPro [41]	http://pepito.proteomics.ics.uci.edu/	Half sphere exposure values
PEPOP [42]	Available upon request	Accessible and sequence contiguous amino acids segments
SEPPA [45]	http://lifecenter.sgst.cn/seppa/index.php	Unit patch of residue triangle
Epitopia [46]	http://epitopia.tau.ac.il/	Based on Naïve Bayes classifier with physicochemical and structural geometrical properties
EPCES [47]	http://sysbio.unl.edu/services/EPCES/	Consensus score by six functions
Shinji Soga^a^ [49]	Not stated	Antibody-specific epitope propensity index
EPSVR & EPMeta [51]	http://sysbio.unl.edu/services/	Based on SVR and meta-analysis
Zhang^a^ [52]	http://code.google.com/p/my-project-bpredictor/downloads/list	Based on random forests with a distance-based feature

^a^The name of the first author is used if the method has no name.

**Table 3:** Available databases for mimotopes data.

Databases	Websites
ASPD [58]	http://wwwmgs.bionet.nsc.ru/mgs/gnw/aspd
RELIC Peptides [59]	Available upon request
PEPBANK [60]	http://pepbank.mgh.harvard.edu
MimoDB [61, 62]	http://immunet.cn/mimodb
Sun's Benchmark datasets [25]	http://cs.nenu.edu.cn/bioinfo/benchmark%20datasets/index.html

**Table 4:** Methods for mimotope-based B-cell epitopes prediction.

Method	Online service (program)	Brief notes
FINDMAP [64]	Not available	Map motif to antigen sequence
EPIMAP [65]	Not available	Improved version of FINDMAP
MIMOP/MimAlign [66]	Available upon request	Based on four multiple sequence alignments
MEPS [67]	http://www.caspur.it/meps	Surface mimicking peptides
3DEX [68]	Not available	Physicochemical neighborhood
MIMOX [88]	http://immunet.cn/mimox/	The first free web tool
MIMOP/MimCons [66]	Available upon request	Clustering the mimotope sequences
Mapitope [69, 70]	http://pepitope.tau.ac.il	The first method based on amino acid pairs
Denisova^a^ [71–73]	Not available	Derivative method of Mapitope
SiteLight [74]	Not available	A patch-based method
EpiSearch [75]	http://curie.utmb.edu/episearch.html	An automated sequence analysis based on sequence and 3D profiles
PepSurf [76]	http://pepitope.tau.ac.il	The first graph-based method
Pep-3D-Search [77]	http://kyc.nenu.edu.cn/Pep3DSearch	Ant colony optimization algorithm
MimoPro [78]	http://informatics.nenu.edu.cn/MimoPro	Based on both patch and graph searching

^a^The name of the first author is used if the method has no name.
